# Contribution of prostanoid signaling to the evolution of spreading depolarization and the associated cerebral blood flow response

**DOI:** 10.1038/srep31402

**Published:** 2016-08-10

**Authors:** Dániel Péter Varga, Tamás Puskás, Ákos Menyhárt, Péter Hertelendy, Dániel Zölei-Szénási, Réka Tóth, Orsolya Ivánkovits-Kiss, Ferenc Bari, Eszter Farkas

**Affiliations:** 1 Department of Medical Physics and Informatics, Faculty of Medicine & Faculty of Science and Informatics, University of Szeged, H-6720 Szeged, Korányi fasor 9, Hungary

## Abstract

The significance of prostanoid signaling in neurovascular coupling during somatosensory stimulation is increasingly more appreciated, yet its involvement in mediating the cerebral blood flow (CBF) response to spreading depolarization (SD) has remained inconclusive. Selective cyclooxygenase (COX) enzyme inhibitors (NS-398, SC-560) or an antagonist (L161,982) of the EP4 type prostaglandin E2 receptor were applied topically to a cranial window over the parietal cortex of isoflurane-anesthetized Sprague-Dawley rats (n = 60). Global forebrain ischemia was induced by occlusion of both common carotid arteries in half of the animals. SDs were triggered by the topical application of 1M KCl. SD occurrence was confirmed by the acquisition of DC potential, and CBF variations were recorded by laser-Doppler flowmetry. EP4 receptor antagonism significantly decreased peak hyperemia and augmented post-SD oligemia in the intact but not in the ischemic cortex. COX-1 inhibition and EP4 receptor blockade markedly delayed repolarization after SD in the ischemic but not in the intact brain. COX-2 inhibition achieved no significant effect on any of the end points taken. The data suggest, that activation of EP4 receptors initiates vasodilation in response to SD in the intact brain, and – together with COX-1 derived prostanoids – shortens SD duration in the acute phase of ischemia.

Recurrent spreading depolarizations (SDs) are slowly propagating waves of electrical silence in the cerebral gray matter^1^ that occur spontaneously in the injured brain^2^,^3^. Recurrent SD events have recently been recognized to exacerbate ischemic brain injury in patients of subarachnoid hemorrhage, stroke or traumatic brain injury^4^, and are being promoted as a causal biomarker assessed in neurocritical care to indicate the degree of metabolic failure in the brain tissue^5^.

SDs are coupled with typical changes in local cerebral blood flow (CBF)^6^. In the rat - and most probably in human - the physiological pattern of the SD-associated CBF response includes four sequential components: (i) an initial, brief hypoperfusion; (ii) a marked, transient peak hyperemia; (iii) a less obvious late hyperemia; and (iv) a sustained hypoperfusion also known as spreading oligemia or post-SD oligemia^6^. The duration and magnitude of these four elements in the CBF response is variable, with the peak hyperemic component being the most conspicuous. In the ischemic brain, the CBF response to SD is more dominated by vasoconstrictive elements, leading to diminishing hyperemia and more prevalent hypoemia^7^,^8^,^9^,^10^. In the most severe form, the hypoemic element completely outweighs hyperemia, and turns into spreading ischemia^11^. This atypical SD-associated CBF variation in the injured brain aggravates metabolic supply-demand mismatch in the tissue, and can delay recovery from SD thereby increasing the risk of irreversible depolarization and neuronal cell death.

The regulation of the SD-related CBF response appears to be rather complex, and the discrimination of any specific individual mediator poses a considerable challenge^6^. In physiological neurovascular coupling during somatosensory stimulation, prostanoids have emerged as potent vasoactive metabolites^12^,^13^. A major pathway leading to vasodilator prostanoid synthesis involves cyclooxygenase-2 (COX-2), a rate limiting, inducible enzyme using arachidonic acid as its substrate. COX-2 is expressed in cortical pyramidal neurons^14^, and is located in perivascular nerve terminals along intraparenchymal penetrating arterioles and capillaries^15^. Most importantly, COX-2 products have emerged as mediators of functional hyperemia to somatosensory stimulation^13^,^16^. A COX-2 derived vasoactive mediator produced by the downstream enzyme prostaglandin E synthase is prostaglandin E2 (PGE_2_)^17^, which causes vasodilation by binding to its receptors (EP2 and EP4 receptors) located in the vascular wall in the brain^13^,^14^,^18^. In contrast with the COX-2 route, the role of the constitutive COX-1 enzyme (which, in the context of physiological neurovascular coupling, is argued to be expressed in astrocytes)^19^ in shaping the CBF response to neuronal activity has remained controversial^19^. Selective COX-1 inhibition blocked the evolution of hyperemia in response to odorant stimulation^20^, or uncaging of Ca^2+^ in perivascular astrocytic endfeet^21^, yet it exerted no impact on the CBF response to whisker stimulation^22^,^23^,^24^.

Arachidonc acid metabolites could possibly play a central role in mediating the CBF response to SD because spreading depolarization coincides with a considerable accumulation of arachidonic acid in the cortex^25^, and a significant elevation of prostanoid concentration (e.g. PGE_2_) in the cerebrospinal fluid^26^. Yet, in contrast with the dominant vasodilator effect of prostaglandins in response to somatonsensory stimulation^13^, arachidonic acid metabolites released due to SD were found to be vasoconstrictive: First, the non-selective inhibition of COX enzymes (i.e. application of indomethacin) caused pial vasodilation with SD, and diminished vasoconstriction underlying the post-SD oligemia^26^, Second, synthesis of the vasoconstrictive hydroxyeicosatetraeonic acid (20-HETE) by the P450 epoxygenase enzyme located in vascular smooth muscle cells was demonstrated in response to SD, and the pharmacological blockade of its synthesis ameliorated the post SD oligemia^27^. Nonetheless, the selective effect of COX-1 or COX-2 products on the SD-associated CBF response has not been revealed, although the potential involvement of COX-2 is conceivable, because COX-2 mRNA and protein were found upregulated in cortical neurons in association with SD^28^.

In summary, even though the significance of the COX-2 pathway in neurovascular coupling during somatosensory stimulation is getting increasingly more appreciated^12^,^13^, its involvement in mediating the CBF response to SD has remained inconclusive and is far from being understood^6^. In addition, COX-2 is remarkably upregulated in the ischemic brain during the period over which deleterious SDs occur spontaneously in rodent ischemia models^29^, but there is essentially no evidence as to whether the ischemia-related COX-2 elevation modulates the CBF response to SD.

Taken together, here we set out to determine systematically whether COX-2 or COX-1 derived metabolites - specifically the vasodilator PGE_2_^18^,^30^ - contribute to the evolution of the SD-coupled hyperemia in the intact or the ischemic rat cerebral cortex. We also sought to assess any potential involvement of prostanoid signaling in the evolution of SD itself.

## Results

### General physiological variables

**Figure 1:**
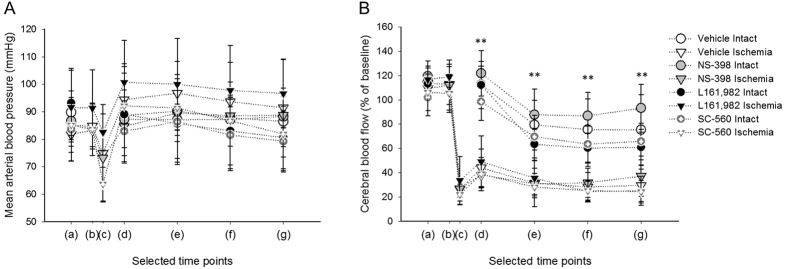
Mean arterial blood pressure (MABP; in (**A**) and drift of local cerebral blood flow (CBF; in (**B**) over the experimental protocol. Selected time points for sampling have been defined as follows: (a) at 35 min after the initiation of pharmacological treatments; (b) immediately before ischemia induction; (c) MABP (**A**) or CBF (**B**) minimum after ischemia induction; (d) prior to SD1; (e) prior to SD2; (f) prior to SD3; (g) prior to SD4. Data are given as mean ± stdev. Statistical analysis relied on a repeated measures paradigm followed by a Fisher post hoc test. Ischemia – but not treatment - had a significant effect on the baseline drift of CBF. Level of significance was determined as **p < 0.01 vs. respective Intact group.

Topical application of drugs was chosen in order to avoid potential systemic side effects. Measured values of mean arterial blood pressure (MABP) and the outcome of blood gas analysis confirmed no difference in the systemic variables assessed in various treatment groups. Specifically, Fig. 1A demonstrates that MABP did not change significantly over the experimental protocol, and was similar across experimental groups. Arterial blood gas analysis showed that all investigated variables were within the physiological range at the start of the experiments (i.e. pO_2_: 133 ± 28 mmHg, pCO_2_: 32 ± 6 mmHg, pH: 7.39 ± 0.02, HCO_3_: 19.19 ± 4.54 mmol/l, glucose: 5.21 ± 1.71 mmol/l) and prior to the termination of the experiments (i.e. pO_2_: 125 ± 29 mmHg, pCO_2_: 38 ± 11 mmHg, pH: 7.37 ± 0.08, HCO_3_: 21.49 ± 3.44 mmol/l, glucose: 6.11 ± 1.92 mmol/l). Baseline CBF was obviously reduced significantly due to ischemia induction (from 112 ± 15 to 27 ± 13% as an average), but the various treatments did not exert any significant impact (Fig. 1B).

### Direct current potential signature of spreading depolarization

**Figure 2:**
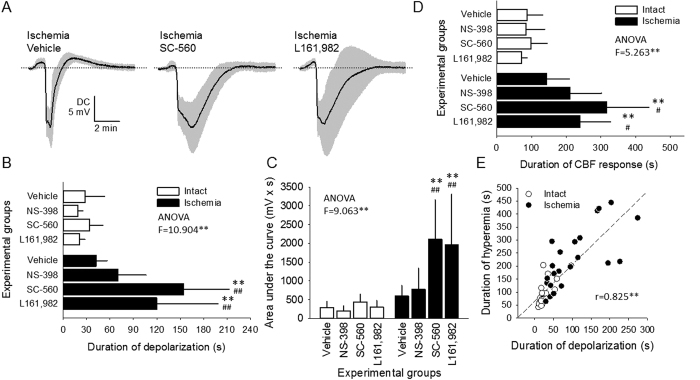
The impact of pharmacological treatments on the kinetics of the spreading depolarization (SD)-related, transient negative shift of the direct current (DC) potential. All panels demonstrate data for recurrent SDs. (**A**) DC potential traces are mean ± stdev, standing for Vehicle, SC-560, and L161,982 treatment under ischemia. (**B**) Duration of the negative shift of the DC potential taken at half amplitude. (**C**) Area under the curve of the negative shift of the DC potential. Note the selective impact of COX-1 inhibition (SC-560) and EP4 receptor blockade (L161,982) under ischemia. (**D**) Duration of the cerebral blood flow (CBF) response to SD taken at half amplitude of peak hyperemia. (**E**) Correlation between the duration of the SD-related negative DC shift and the associated CBF response; all groups are shown. Data shown in Panels B-D are given as mean ± stdev. Statistical analysis relied on a one-way analysis of variance (ANOVA) model followed by a Fisher post hoc test. Statistical significance was determined as **p < 0.01 vs. respective Ischemia, and ^#^p < 0.05 or ^##^p < 0.01 vs. respective Vehicle. The correlation presented in Panel E was tested with a Pearson one-tailed correlation analysis (**p < 0.01).

Pharmacological manipulation proved to be ineffective on the kinetics of SD in the Intact groups. Conversely, the DC potential signature of SD was markedly and selectively altered by SC-560 and L161,982 treatment in the Ischemic group (Fig. 2A). As such, the duration of the negative DC shift at half amplitude was substantially increased for both the first SD (117 ± 32 and 95 ± 64, vs. 43 ± 15 s; Ischemic SC-560 and Ischemic L161,982 vs. Ischemic Vehicle), and recurrent SDs (154 ± 58 and 120 ± 79, vs. 42 ± 14 s; Ischemic SC-560 and Ischemic L161,982 vs. Ischemic Vehicle) (Fig. 2B). Similarly, the magnitude of the DC shift characterized by the area under the curve greatly increased due to SC-560 and L161,982 treatment in combination with ischemia, for both the first SD (1499 ± 340 and 1478 ± 1263, vs. 574 ± 384 mV x s; Ischemic SC-560 and Ischemic L161,982 vs. Ischemic Vehicle), and recurrent SDs (2104 ± 1056 and 1958 ± 1357, vs. 598 ± 281 mV x s; Ischemic SC-560 and Ischemic L161,982 vs. Ischemic Vehicle) (Fig. 2C). In addition, SC-560 and L161,982 treatment markedly reduced the amplitude of post-SD hyperpolarization (e.g. recurrent SDs: 1.16 ± 0.50 and 1.40 ± 0.62, vs. 2.65 ± 0.39 mV; Ischemic SC-560 and Ischemic L161,982 vs. Ischemic Vehicle), which also concerned only the ischemic animals but not the intact ones. In contrast, NS-398 treatment proved to be statistically ineffective for all the above variables as compared with Vehicle.

### Cerebral blood flow response to spreading depolarization

In contrast with the analysis of the DC potential signature of SD, the impact of pharmacological treatments on the CBF response could be discriminated in the intact – but not in the ischemic - animals. The selective COX enzyme inhibitors NS-398 and SC-560 did not exert any clear influence on the evolution of the SD-related CBF response, as all the examined variables remained unaltered by the treatments.

**Figure 3:**
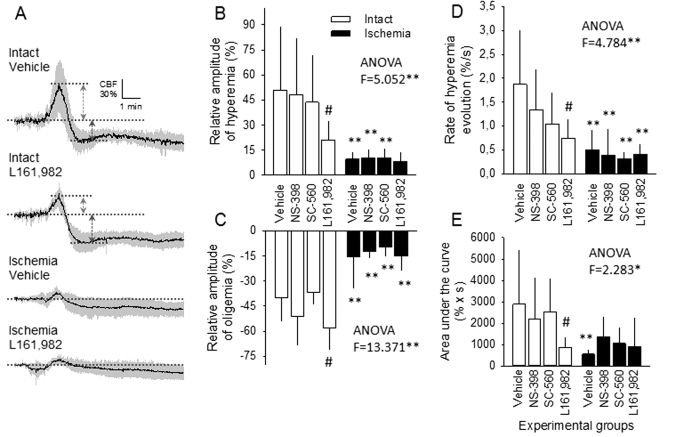
The impact of pharmacological treatments on the kinetics of the spreading depolarization (SD)-related cerebral blood flow (CBF) response. All panels demonstrate data for the first SD. A, CBF traces are mean ± stdev for each group presented. (**B**) Maximum amplitude of peak hyperemia; (**C**) Minimum amplitude of post-SD oligemia; (**D**), Rate of hyperemia evolution associated with SD; (**E)**, Magnitude of hyperemia expressed as area under the curve. Data in Panels B-E are given as mean ± stdev. Statistical analysis relied on a one-way analysis of variance (ANOVA) model followed by a Fisher post hoc test. Statistical significance was determined as *p < 0.05 and **p < 0.01 vs. respective Intact, and ^#^p< 0.05 vs. respective Vehicle. Note the selective effect of L161,982 (i.e. EP4 receptor antagonism) under Intact condition.

On the other hand, the EP4 receptor blocker L161,982 selectively reduced the relative amplitude of peak hyperemia with the first SD (21 ± 11 vs. 51 ± 38%, Intact L161,982 vs. Intact Vehicle), and recurrent SDs (50 ± 21 vs. 76 ± 37%, Intact L161,982 vs. Intact Vehicle). In fact, L161,982 lowered the amplitude of the hyperemic element of the CBF response to near the level observed for the ischemic group (first SD: 21 ± 11 vs. 15 ± 8%, L161,982 Intact vs. L161,982 Ischemic), as indicated by the loss of statistically significant difference otherwise obvious between the intact-ischemic group pairs (i.e. Vehicle, NS-398 or SC-560 treated) (Fig. 3B). Further, L161,982 augmented the relative amplitude of post-SD oligemia with the first SD (58 ± 13 vs. 40 ± 14%, Intact L161,982 vs. Intact Vehicle) (Fig. 3A,C). Based on these results, the selective EP4 receptor blocker L161,982 appeared to achieve a general loss of a dilatory element prevalent throughout the CBF response, as reflected by a reduction of peak hyperemia with a degree matching the deepening of the post-SD oligemia (Fig. 3A). This observation was confirmed by calculating the flow difference between peak hyperemia and the minimum point of post-SD oligemia, providing the exploited range of vasoregulation during the CBF response, which was unchanged due to treatments in the intact groups (87 ± 32 pp, % in average).

To further prove the selective effect of L161,982 on the SD-related CBF response, hyperemia evolution significantly decelerated due to L161,982 treatment as demonstrated by its shallower upward slope with the first SD (0.75 ± 0.38 vs. 1.88 ± 1.13%/s, Intact L161,982 vs. Intact Vehicle) (Fig. 3D), and recurrent SDs (1.05 ± 0.43 vs. 1.60 ± 0.70%/s, Intact L161,982 vs. Intact Vehicle). Finally, the magnitude of hyperemia expressed as the area under the curve was also significantly decreased by L161,982 for the first SD (874 ± 462 vs. 2885 ± 2543% x s, Intact L161,982 vs. Intact Vehicle) (Fig. 3E), and recurrent SDs (5532 ± 3643 vs. 7448 ± 2072% x s, Intact L161,982 vs. Intact Vehicle).

The duration of the hyperemic element of the CBF response appeared to deserve separate consideration, as the changes due to pharmacological treatments seemed to be consistent with the results derived from the DC potential signature of SD (Fig. 2), rather than other variables assessed for the CBF response (Fig. 3). Indeed, the duration of hyperemia markedly elongated in the ischemic animals, especially due to treatment with SC-560 and L161,982 (e.g. recurrent SDs: 316 ± 122 and 240 ± 89 vs. 143 ± 67 s; Ischemic SC-560 and Ischemic L161,982 vs. Ischemic Vehicle) (Fig. 2D). The coincidence between the duration of depolarization and the associated hyperemia was confirmed by a strong, positive correlation (r = 0.825**) between these variables (Fig. 2E).

Finally, the early hypoperfusion element of the SD-related CBF response did not appear consistently, therefore measured values (i.e. amplitude, duration) could not be analyzed reliably.

### The impact of L161,982 treatment on ischemia outcome

In addition to its vasoconstrictive potential as detailed above, L161,982 administration caused the greatest deviation in all assessed variables, and contributed to the evolution of severe ischemia in a third of the animals assigned to L161,982 treatment (Table 1). Such severe ischemia did not occur in any of the other treatment groups. The most obvious signs of the adverse effect of L161,982 administration were (i) the occurrence of spontaneous SDs within minutes after ischemia onset - and their long duration, (ii) the inability of the tissue to sustain subsequent, triggered SDs, (iii) the lack of detectable CBF response to these SDs, (iv) lower CBF levels at all time points after ischemia onset as compared with any other groups, and (v) occasional, lethal outcome before the termination of the experimental protocol (n = 2).

## Discussion

**Figure 4:**
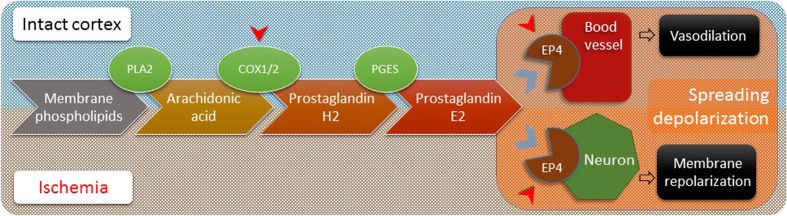
Schematic illustration of the metabolic signaling pathway targeted in the present study, and its suggested involvement in modulating spreading depolarization and the related cerebral blood flow response. Red arrowheads indicate the sites of pharmacological interventions used. Abbreviations: PLA2, phospholipase A2; COX, cyclooxygenase; EP4, receptor type for prostaglandin E_2_; PGES, prostaglandin E synthase.

Here we set out to discriminate the potential involvement of prostanoids, particularly PGE_2_ in the regulation of the CBF response to SD. Two experimental approaches have been utilized. First, we selectively inhibited the COX-1 and COX-2 enzymes, which produce the precursor for PGE_2_ synthesis and thereby regulate the availability of PGE_2_ (Fig. 4). In support of this approach, the inhibition of COX-2 was previously shown to significantly reduce functional hyperemia in response to whisker stimulation^16^. Next, we pharmacologically blocked the EP4 receptors of PGE_2_ (Fig. 4), because EP4 receptors expressed in cerebral arteries were shown to mediate the vasodilatory effect of PGE_2_^18^,^30^, and their activation contributed to functional hyperemia in response to somatosensory stimulation^14^. Because PGE_2_ synthetized via COX-2 suppresses neuronal excitability^31^ and the COX-2 - EP4 signaling pathway has been implicated in the progression of ischemic injury^32^,^33^, we assessed the impact of the chosen pharmacological treatments on the DC signature of SD, as well. Finally, the neuronal or vascular upregulation of COX-2 and EP4 receptors established in cerebral ischemia^29^,^33^ prompted us to investigate whether the kinetics of SD or the related CBF response are modulated by this signaling pathway in the ischemic brain. This is particularly relevant, as spontaneous, recurrent SDs have been implicated in the progression of ischemic injury^2^,^3^,^4^,^5^.

Of the subsequent phases of the CBF response to SD^6^, we focused on the peak hyperemic and post-SD oligemic elements. The first significant observation of the current study is that EP4 receptor antagonism reduced peak hyperemia, and augmented post-SD oligemia of the CBF response to SD in the intact brain (Fig. 3B,C). This indicates that EP4 receptor activation contributes to vasodilation during the CBF response to SD (Fig. 4). It is a novel finding, as the mediation of the hyperemic element of the CBF response by prostanoids was previously thought unlikely, and COX-derived metabolites were attributed a vasoconstrictive rather than a vasodilatory role in the CBF response to SD^6^,^26^. At the same time, our data are consistent with a number of previous reports on the vasodilatory action of EP4 receptor activation by PGE_2_. For example, antagonism of EP4 receptors inhibited the PGE_2_-induced vasodilation of the middle meningeal artery of rats *in vivo*, and of the middle cerebral artery *in vitro*^18^,^30^. At the concentration used here, L161,982 previously reduced the NMDA-induced dilation of cerebral arterioles, functional hyperemia to whisker stimulation^14^, and glutamate-evoked dilation of microvessels^34^. Our data thus indicate that EP4 receptor activation achieves CBF elevation in response to SD, as well.

The selective inhibition of COX-1 and COX-2 enzymes exerted no significant, direct effect on any of the elements of the CBF response to SD (Fig. 3), despite the expectation that COX-2 inhibition by NS-398 would decrease the magnitude of the hyperemic element as it did functional hyperemia in response to whisker stimulation previously^16^. The reason for the absence of the COX-enzyme related vasodilation may well be that during SD, the activity of COX enzymes also leads to the marked production of vasoconstrictive prostanoids, such as prostaglandin F2α^26^. Inhibition of the COX enzymes as was done in our experiments therefore likely diminishes the SD-related synthesis of both vasoconstrictive and vasodilator prostanoids, causing an undiscernible net outcome on the CBF response. The results of others and of ours together show, that functional hyperemia to neuronal activation is COX-2 dependent (i.e. selective COX-2 inhibition alone reduces the peak of hyperemia)^14^,^16^,^23^, while COX-2 inhibition has no clear impact on the CBF response to SD. These data foster the assumption that the CBF response to SD is driven by mechanisms different from physiological neurovascular coupling. Taken prostanoid-based cerebral vasoregulation, it is thus conceivable that vasoconstrictive COX products are released at insignificant concentration during neuronal activation, but their impact is substantial during SD^26^, obscuring the co-existing vasodilator effect of PGE_2_.

In the ischemic cortex, our study revealed a considerable reduction of the distinct elements of the CBF response to SD, with no detectable impact of COX enzyme inhibition or EP4 receptor blockade. Consistent with the data presented here, ischemia is known to impair physiological neurovascular coupling as evidenced by the attenuation of functional hyperemia to forepaw stimulation^35^. In addition, in the acute phase of ischemia (the target of investigation here), the abundant release of metabolic mediators of vascular tone, such as adenosine, increased ADP/ATP ratio, lactate accumulation, or the dramatically elevated concentration of extracellular potassium^36^ must obscure potentially still effective, finer signals of vasoregulation. It may also well be that the prostanoid-based regulation of vascular tone becomes more obvious and could prove discernable during the subacute or chronic phases of ischemia, because COX-2 mRNA expression becomes upregulated beginning 6 hours, and endothelial EP4 receptor expression is induced 4 hours after ischemia onset^29^,^37^.

In summary, EP4 receptor activation contributes to vasodilation during the CBF response to SD in the intact cortex, and is undetectable under the acute phase of ischemia, possibly due to impaired neurovascular coupling, the dominance of metabolic mediators of vascular tone, or the upregulation of the receptors only several hours later.

The present study revealed that the selective pharmacological inhibition of COX-1 or the antagonism of EP4 receptors in the ischemic cortex both hamper the recovery of the nervous tissue from SD (as indicated by increased SD duration, Fig. 2A,B), while the inhibition of COX-2 remained ineffective in this respect. It has been firmly established that COX-2 expression is markedly upregulated during cerebral ischemia, and COX-2 inhibition is neuroprotective - despite cardiovascular side effects^38^. In our hands, COX-2 inhibition was ineffective on the neurophysiological end points taken, most certainly because in this early phase of ischemia COX-2 was not yet upregulated^29^. Instead, the role of COX-1 in promoting repolarization after SD has emerged. These results of ours stand in harmony with the report that ischemia-related hippocampal PGE_2_ production in a gerbil ischemia model was potently reduced by COX-1 rather than COX-2 inhibition during the early phase of reperfusion in contrast with 24 hours later, when COX-2 appeared to be the dominant source of PGE_2_^39^. Despite the daunting complexity of prostanoid signaling, it is tempting to speculate, that COX-1 derived PGE_2_ should activate neuronal EP4 receptors during SD in the acute phase of ischemia, and thereby promote neuronal repolarization (Fig. 4). This would be an effective means of neuronal survival consistent with previous data demonstrating that longer SD duration predicts more extensive ischemic injury^40^ and genetic inactivation of neuronal EP4 receptors worsens stroke outcome^33^. In fact, shortened SD duration mediated by neuronal EP4 receptors could possibly account for smaller infarct volume reported to be achieved by selective EP4 receptor agonism^33^,^41^.

Even though our results focus on EP4 receptors, PGE_2_ was shown to achieve neuroprotection by activating the EP2^42^ and possibly the EP3 receptors, although there is no consensus on whether EP3 receptor activation is protective under all circumstances^43^,^44^. It is therefore plausible that COX-1 derived PGE_2_ could achieve the shortening of SD duration through EP2 and perhaps EP3 receptors, as well.

In the ischemic cortex, the duration of hyperemia in response to SD increased synchronously with elongated SD duration imposed by COX-1 inhibition or EP4 receptor antagonism (Fig. 2B,D). The synchronicity was confirmed by the strong positive correlation between SD and hyperemia duration (Fig. 2E). Earlier we postulated based on similar observations that the return of CBF to baseline after hyperemia may be postponed by the continuing energy need to restore membrane potential^10^. For this reason, it is conceivable that increased hyperemia duration seen as a result of COX-1 inhibition or EP4 receptor antagonism must have been achieved indirectly, by increasing the duration of SD itself.

Here we applied the EP4 receptor blocker L161,982 in a concentration that weakens neurovascular coupling in the intact nervous tissue^14^,^34^. In addition to our present results that EP4 receptor antagonism delayed tissue recovery after SD during ischemia (see above), we found a major, detrimental impact of EP4 receptor antagonism on ischemia outcome in a subpopulation of animals assigned to the treatment (Table 1). The symptoms revealed both vascular and neurophysiological impairment (i.e. spontaneous SD occurrence with no detectable CBF response, markedly reduced baseline CBF during ischemia) indicative of the interaction of these elements, or the potential involvement of both neuronal and vascular EP4 receptors^32^,^33^. Our data confirm the notion that EP4 receptor activation is necessary for neuronal survival and better ischemia outcome as previously shown by methods including agonism of EP4 receptors, EP4 receptor deletion, and the assessment of EP4 receptor expression in various experimental models of cerebral ischemia^32^,^33^,^41^. Even though our data are thus consistent with previous reports, the severe outcome due to L161,982 treatment was unexpected, as neurovascular coupling studies reported no such risk^14^,^34^ and no previous investigations have evaluated the impact of L161,982 administration on neurophysiological outcome after ischemia. Finally, we suggest that SD evolution may be a critical component of infarct development related to the inactivation of EP4 receptors, as implied by the spontaneous occurrence of long-lasting SD in these animals.

The restriction of SD evolution in neurological intensive care patients has become a central objective, which is driven by the recognition that spontaneously occurring recurrent SDs of long cumulative duration worsen the outcome of ischemic or traumatic brain injury^4^,^5^. So far, the inhibition of NMDA receptors by ketamine administration has been the only strategy of promise to decrease the likelihood of SD occurrence^45^. PGE_2_ and its specific receptors have long been implicated in mediating neuronal death or survival under ischemia, but have not been associated with SD evolution. The data presented here demonstrate for the first time that the inhibition of COX-1 or the blockade of the EP4 receptors of PGE_2_ remarkably increases SD duration in the early phase of cerebral ischemia. These significant observations may initiate a new line of investigation to dissect specific components of prostanoid signaling that may play a defining role in sustaining or aborting SD in the ischemic nervous tissue. In the long run, specific elements of prostanoid signaling may present new, effective targets in addition to NMDA receptors to restrict SD evolution and thereby lessen SD-related injury.

## Materials and Methods

The experimental procedures were approved by the National Food Chain Safety and Animal Health Directorate of Csongrád County, Hungary. The procedures conformed to the guidelines of the Scientific Committee of Animal Experimentation of the Hungarian Academy of Sciences (updated Law and Regulations on Animal Protection: 40/2013. (II. 14.) Gov. of Hungary), following the EU Directive 2010/63/EU on the protection of animals used for scientific purposes, and reported in compliance with the ARRIVE guidelines.

### Surgical procedures

Surgical procedures were similar to what was previously described^46^. Young adult male Sprague-Dawley rats (n = 60, body weight: 302 ± 31 g) were used in the study. The animals were purchased from the Charles River Laboratories, Hungary, were group-housed under a normal 12/12 h light/dark cycle, and had free access to food and drinking water. On the day of experiments, the animals were anesthetized with 1.5–2% isoflurane in N_2_O:O_2_ (70%:30%), and breathed spontaneously through a head mask throughout the experiment. Body temperature was maintained at 37 °C with a servo-regulated heating pad. Atropine (0.1%, 0.05 ml) was administered intramuscularly shortly before surgical procedures to avoid the production of airway mucus. A catheter was inserted into the left femoral artery to monitor mean arterial blood pressure (MABP) continuously, and arterial blood gas levels at the start of each experimental protocol, and before termination of the experiments.

Next, a midline incision was made in the neck and both common carotid arteries were delicately separated from the surrounding muscle and the vagal nerves. Lidocain (1%) was administered topically before opening each tissue layer. A silicone coated fishing line used as occluder was looped around each artery for later induction of cerebral ischemia. Rats were transferred to a stereotactic frame and fixed in prone position. Two craniotomies (5 mm lateral from midline, −3 mm and −7 mm caudal from bregma) were prepared over the right parietal cortex using a dental drill (Technobox, Bien Air 810). A doughnut shape ring of acrylic dental cement was built around the rostral craniotomy for the latter topical application of drugs to the cortical surface. The dura in each craniotomy was carefully dissected, and the craniotomies were regularly irrigated with artificial cerebrospinal fluid (aCSF; mM concentrations: 126.6 NaCl, 3 KCl, 1.5 CaCl_2_, 1.2 MgCl, 24.5 NaHCO_3_, 6.7 urea, 3.7 glucose bubbled with 95% O_2_ and 5% CO_2_ to achieve a constant pH of 7.4). In the rostral window, an intracortical glass capillary microelectrode (outer tip diameter = 20 μm) was lowered 800–1000 μm into the cortex, and an Ag/AgCl electrode was implanted under the skin of the animal’s neck to be used as reference. In order to assess changes in local CBF, a laser-Doppler needle probe (Probe 402 or 403 connected to Perimed 4001 Master or PeriFlux 5000; Perimed AB, Sweden) was positioned near the cortical surface, adjacent to the intracortical microelectrode, avoiding any large pial vessels. The caudal craniotomoy was later used for SD elicitation.

### Electrophysiology and cerebral blood flow measurement

Direct current (DC) potential was recorded via a high input impedance pre-amplifier (NL102G or NL100AK, NeuroLog System, Digitimer Ltd, United Kingdom), connected to a differential amplifier (NL106 or NL107, NeuroLog System, Digitimer Ltd, United Kingdom) with associated filter and conditioner systems (NL125, NL144 or NL530, NeuroLog System, Digitimer Ltd, United Kingdom). Potential line frequency noise (50 Hz) was removed by a high quality noise eliminator (HumBug, Quest Scientific Instruments Inc., Canada) without any signal attenuation. The resulting signal was digitalized either by an analog/digital (A/D) converter (MP150, Biopac Systems Inc., USA) and continuously acquired at a sampling frequency of 1 kHz or 500 Hz using the software AcqKnowledge 4.2.0 (Biopac Systems Inc., USA), or another dedicated A/D converter card (NI USB-6008/6009, National Instruments, Austin, Texas, USA) controlled through a custom-made software, written in Labview (National Instruments, Austin, Texas, USA). The laser Doppler signal was digitized and acquired, together with the DC potential, essentially as described above.

### Drug administration

Drug solutions or vehicle of equal volume (1.5% DMSO in 10 ml aCSF) were superfused on the cortical surface free of dura in the rostral cranial window after taking DC potential and CBF baseline for 5 min under aCSF. Drug concentrations were carefully selected based on dose response curves, selectivity and efficacy reported previously^14^,^16^,^18^,^34^. The following drugs were applied topically: the selective COX-2 inhibitor NS-398 (100 μM; Cayman)^16^, the selective COX-1 inhibitor SC-560 (25 μM; Cayman)^22^, or the selective PGE_2_ receptor (EP4) antagonist L161,982 (1 μM; Sigma)^14^,^34^. The pharmacological treatment was initiated 40 min prior ischemia induction or the elicitation of the first SD event (i.e. sham-operated group)^16^ and incubation persisted till the end of the experimental protocol.

### Ischemia induction and SD elicitation

Following the 40-min incubation period, persistent incomplete global forebrain ischemia was induced in half of the animals, by occluding both common carotid arteries permanently (“2-vessel occlusion”, 2VO): occluders were pulled on until resistance was felt and then secured in place. Successful ischemia induction was confirmed by a sharp drop of the laser Doppler signal. As control for ischemia, the remaining animals were used as a sham-operated group (SHAM), in which the occluders were not pulled but left in place.

Forty minutes after the start of drug incubation, 4 SDs with an inter-SD interval of 15 minutes were triggered by placing a 1 M KCl-soaked cotton ball on the exposed cortical surface in the caudal cranial window. The cotton ball was removed and the caudal cranial window rinsed with aCSF immediately after each successful SD elicitation. Experiments were terminated by the overdose of the anesthetic agent, 20 min after triggering the last SD. The combination of pharmacological treatment and ischemia induction resulted in 8 experimental groups (Table 2).

### Data analysis

All variables (i.e. DC potential, CBF and MABP) were simultaneously acquired, displayed live, and stored using a personal computer equipped with a dedicated software (AcqKnowledge 4.2 for MP 150, Biopac Systems, Inc., USA). Data analysis was assisted by the inbuilt tools of the same software, or was transferred into a MATLAB environment (MathWorks Inc., USA). Both DC potential and CBF recordings were first downsampled to 1 Hz. The transient negative DC shift indicative of SD was analyzed to assess: amplitude of depolarization, duration of depolarization at half amplitude, area under the curve of the negative DC shift, slope of depolarization, slope of repolarization, and amplitude of post-SD hyperpolarization.

Raw CBF recordings were expressed relative to baseline by using the average CBF value of the first 5 min under aCSF (100%) and the recorded biological zero obtained after terminating the experiment (0%) as reference points. Drifting of baseline was determined by sampling a 5-min average 30–35 min after the start of drug incubation, a 10 s average prior ischemia induction and during the drop after ischemia induction, and 5-min averages prior each SD elicited. The following elements of the SD-related CBF response were characterized: amplitude and duration of early hypoperfusion, amplitude and duration of hyperemia, magnitude of hyperemia (area under the curve), and the amplitude of post-SD oligemia.

The data were evaluated separately for the first SD, and subsequent, recurrent SDs, because of the obvious differences in the kinetics of the SD-associated CBF response. Data of the 3 recurrent SDs were averaged for each animal, thus a single value per animal of each read-out was taken for statistical analysis.

Data are given as mean ± stdev. The software SPSS (IBM SPSS Statistics for Windows, Version 22.0, IBM Corp.) was used for statistical analysis. A one-way analysis of variance (ANOVA) model was applied for the evaluation of variables derived from the DC potential signature of SD, and the associated CBF response. A repeated measures paradigm was used to evaluate MABP variation and CBF baseline drift. Correlation analysis relied on a one-tailed Pearson test. Levels of significance were defined as p < 0.05* and p < 0.01**.

## Additional Information

**How to cite this article**: Varga, D. P. *et al*. Contribution of prostanoid signaling to the evolution of spreading depolarization and the associated cerebral blood flow response. *Sci. Rep*. **6**, 31402; doi: 10.1038/srep31402 (2016).

## Figures and Tables

**Table 1:** Two distinct subpopulations of experiments carried out to assess the impact of L161,982 treatment (EP4 receptor antagonism) on SD evolution during ischemia.

	Experimental protocol completed (n = 8)	Experimental protocol aborted (n = 4)
Minimum CBF after ischemia onset (%)	33.68 ± 19.75	12.32 ± 6.96
Occurrence of spontaneous SD	no (0/8)	yes (3/4)
Duration of first evoked SD (completed protocol) or spontaneous SD (aborted protocol) (s)	95 ± 64	520 ± 328
Successful elicitation of subsequent recurrent SDs	yes	no
Type of CBF response to first evoked SD (completed protocol) or spontaneous SD (aborted protocol)	hyperemic	no detectable CBF response
CBF prior to the termination of the experiment (%)	25.11 ± 15.00	15.10 ± 10.90

Data are given as mean ± stdev. Abbreviations: CBF, cerebral blood flow; SD, spreading depolarization.

**Table 2:** Composition of experimental groups.

Experimental group	Pharmacological treatment	Ischemia induction (SHAM or 2VO)	n
1	Vehicle (1.5% DMSO in aCSF)	SHAM	8
2		2VO	7
3	100 μM NS-398 (COX-2 inhibition)	SHAM	7
4		2VO	6
5	25 μM SC-560 (COX-1 inhibition)	SHAM	7
6		2VO	7
7	1 μM L161,982 (EP4 receptor antagonism)	SHAM	6
8		2VO	12

Abbreviations: 2VO, permanent, bilateral common carotid artery occlusion (“two-vessel occlusion”); COX, cyclooxygenase enzyme; DMSO, dimethyl sulfoxide; EP4, receptor for prostaglandin E2; SHAM, sham-operated control for ischemia.
